# Oct4 Targets Regulatory Nodes to Modulate Stem Cell Function

**DOI:** 10.1371/journal.pone.0000553

**Published:** 2007-06-20

**Authors:** Pearl A. Campbell, Carolina Perez-Iratxeta, Miguel A. Andrade-Navarro, Michael A. Rudnicki

**Affiliations:** 1 Sprott Centre for Stem Cell Research, Ottawa Health Research Institute, Ottawa, Ontario, Canada; 2 University of Ottawa, Department of Cellular and Molecular Medicine, Ottawa, Ontario, Canada; Max Planck Institute for Evolutionary Anthropology, Germany

## Abstract

Stem cells are characterized by two defining features, the ability to self-renew and to differentiate into highly specialized cell types. The POU homeodomain transcription factor *Oct4* (*Pou5f1*) is an essential mediator of the embryonic stem cell state and has been implicated in lineage specific differentiation, adult stem cell identity, and cancer. Recent description of the regulatory networks which maintain ‘ES’ have highlighted a dual role for *Oct4* in the transcriptional activation of genes required to maintain self-renewal and pluripotency while concomitantly repressing genes which facilitate lineage specific differentiation. However, the molecular mechanism by which *Oct4* mediates differential activation or repression at these loci to either maintain stem cell identity or facilitate the emergence of alternate transcriptional programs required for the realization of lineage remains to be elucidated. To further investigate *Oct4* function, we employed gene expression profiling together with a robust statistical analysis to identify genes highly correlated to *Oct4*. Gene Ontology analysis to categorize overrepresented genes has led to the identification of themes which may prove essential to stem cell identity, including chromatin structure, nuclear architecture, cell cycle control, DNA repair, and apoptosis. Our experiments have identified previously unappreciated roles for *Oct4* for firstly, regulating chromatin structure in a state consistent with self-renewal and pluripotency, and secondly, facilitating the expression of genes that keeps the cell poised to respond to cues that lead to differentiation. Together, these data define the mechanism by which *Oct4* orchestrates cellular regulatory pathways to enforce the stem cell state and provides important insight into stem cell function and cancer.

## Introduction

Embryonic Stem Cells (ESCs) are derived from the inner cell mass of the pre-implantation embryo and are characterized by their unlimited capacity for self-renewal and their ability to contribute to all cell lineages. The successful derivation and culture of human ESCs (hESCs) [1] has opened the possibility of their use for generating cells for transplant, for tissue engineering or for drug development and testing. Importantly, full exploitation of the potential of hESCs will require the complete understanding of the function of the genetic factors that specify stem cell identity and regulate their commitment towards specific differentiated cell lineages. However, the transcriptional networks and molecular mechanisms that regulate the formation, self-renewal, and differentiation of hESC and mouse ESC (mESC) remain at best poorly understood.

*Oct4 (Pou5f1)*, a POU-homeodomain transcription factor, plays a central role in self-renewal, pluripotency, and lineage commitment. Initially expressed as a maternal transcript, *Oct4* is required for the formation of a pluripotent inner cell mass [2]. Moreover, strict control of *Oct4* expression is necessary to maintain ESC identity. Alterations in Oct4 expression promote differentiation and leads to the specification of ectodermal [3], endodermal [4], or mesodermal [5] primitive progenitors. Furthermore, *Oct4* has been shown to promote tumor growth in a dose dependent manner [6] and epithelial dysplasia by interfering with progenitor cell differentiation [7], is expressed in various human tumors [8], [9] and adult stem cells [10] thus extending the role of *Oct4* from embryo to adult.

Recent identification of *Oct4* transcriptional targets in ESCs has revealed an unanticipated collaboration between *Oct4*, Sox2, and Nanog and provides a starting framework of the core transcriptional circuitry which maintains ‘ES’ through coordination of a series of feedback and feedforward loops [11], [12]. Furthermore, several signaling pathways including LIF/JAK/STAT, BMP, WNT, PI3K, MAPK/ERK, TGFβ and Notch [13], [14], [15], [16] have been shown to modulate stem cell function. Several key questions however still remain unresolved as a result of these studies. Firstly, what are the regulatory mechanisms that maintain self-renewal and pluripotency? Conversely, what are the molecular inputs that drive differentiation? Finally, and most importantly, can we deduce the essential themes that characterize stem cell function and thereby utilize this knowledge to gain insight into normal developmental processes to predict the consequences of aberrations to these processes that ultimately lead to human disease?

To address these questions and further elucidate the factors that mediate stem cell function, we undertook an analysis to identify genes whose expression is correlated to Oct4. With the understanding that coexpression of genes may imply coregulation and participation in similar biological processes [17], we sought to identify genes which were correlated to Oct4 transcript expression in a wide variety of stem/progenitor populations which were analyzed by Affymetrix GeneChip technology as part of the Stem Cell Genomics Project [18]. We hypothesized that by using Oct4 as a marker gene for self-renewal, pluripotency, and early lineage commitment, this analysis would lead to the identification of 1) Genes that are central to stem cell identity; 2) *Oct4* target genes; and 3) Genes that modulate *Oct4* function. Although several previous studies have sought to harmonize our understanding of ‘stemness’ [19], [20] it has been suggested that rather than the capacity for self-renewal and differentiation, the unique defining feature of a stem cell is that it represents a lasting steady-state of gene expression suspended in its differentiation pathway, yet maintaining the ability to respond to niche induced signals to carry out the indicated program of cellular specialization [21]. Insight into the juncture between cell extrinsic and intrinsic factors described above will provide an enhanced understanding of the molecular mechanisms which confer stem cells with this ability.

Lineage commitment can be described as a process whereby the unlimited ability for self-renewal and potency are gradually restricted as a cell progresses from one steady state of gene expression to the next. Recently attributed to stochastic events which increase the likelihood of a specific developmental outcome [22], this view is in direct opposition to determinism, which precludes the processing of molecular cues emanating from the cellular niche. In juxtaposition to both the stochastic and deterministic models of development is the view that cellular commitment is facilitated by a hierarchy of transcriptional regulatory networks [23] which exert precise biological control by combinatorial interactions at the protein-protein, and protein-DNA level. The function of these networks is highly responsive to molecular inputs, allowing the rapid processing and relay of information required for either maintenance of a specific cellular state, or progression to an altered steady state. Importantly, our data suggests that Oct4 maintains stem cell identity by targeting key regulatory genes which play critical roles in determining cell fate.

## Results and Discussion

### Oct4 Correlation Analysis

A set of 45 murine samples collected as part of the *Stem Cell Genomics Project* and deposited in *StemBase* **(** **http://www.StemBase.ca/** **)** [18] were selected to form the basis of this analysis (Supplemental Table S1). A wide variety of samples comprising adult and embryonic stem cells and their differentiated derivatives were collected in biological triplicate and hybridized to the Affymetrix MOE430 GeneChip Set for a total of 270 GeneChips. Following normalization, scaling, and filtering of the data the standard Pearson correlation coefficient (rho) between every probeset which passed the filter, to the *Oct4* probeset was computed. A probeset was considered correlated to *Oct4* if |rho|≥0.75. This computation was repeated 10,000 times with random subsets consisting of 65% to 70% of the data. Probesets that were correlated to the Oct4 associated probe in at least 40% of the trials were retained for further analysis (Supplemental Table S2).

The stringency of our correlation analysis is set by two parameters; |rho|≥0.75 and the percentage of trials in which this value for rho is met or exceeded. In setting these parameters our aim was to prioritize genes for analysis which may have either represented *Oct4* targets or genes which were implicated in self-renewal, pluripotency, or early lineage commitment. The values were pragmatic in nature; chosen as such to produce a reasonable number of genes which could be analyzed in a coherent fashion, possibly being able to provide a snapshot as it were of ‘stemness’. The use of more or less stringent parameters would result in the identification of fewer or more genes. Of note, cursory examination of the cutoffs used reveals that should we have increased the percentage of trials for which |rho|≥0.75 from 40% to 50% we would not have identified at least two previously identified Oct4 targets; Sox2 (49%) and Cdyl (40%) [11], [12].

**Figure 1:**
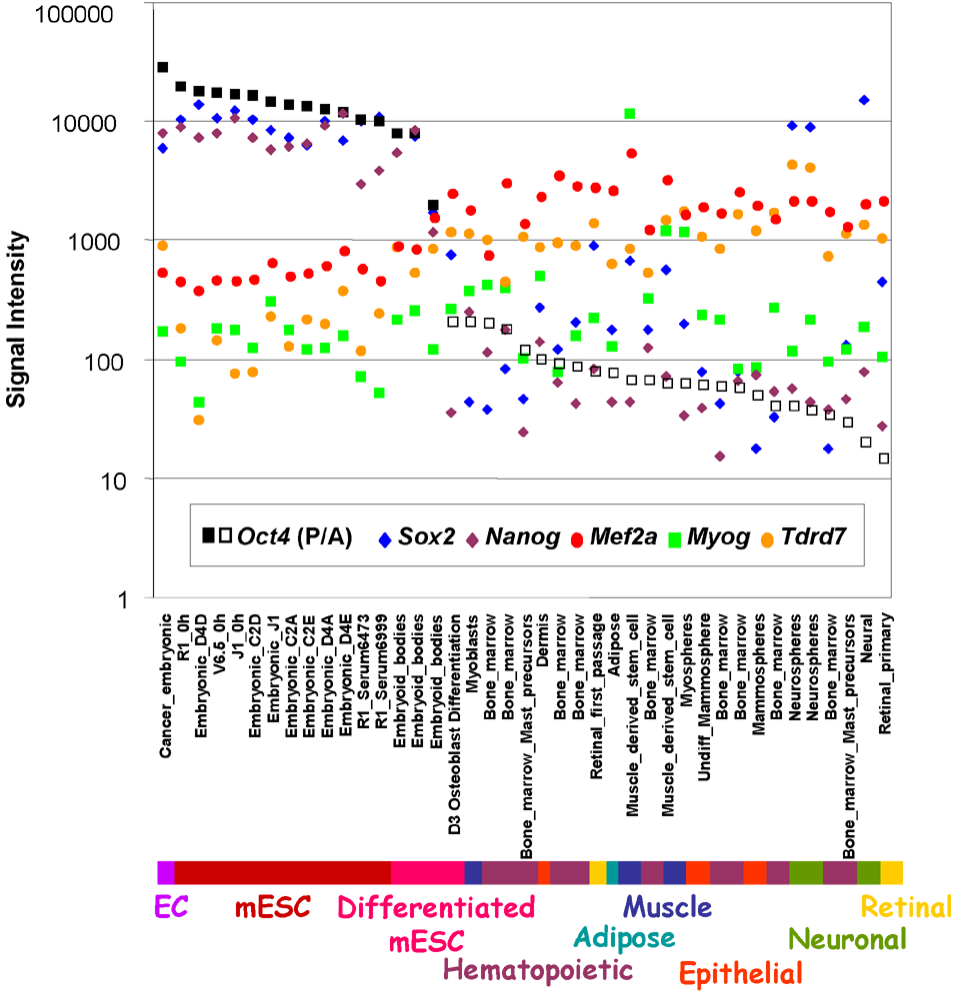
Oct4 Correlation Analysis. ESC, EC, myogenic, neuronal, retinal, and hematopoietic stem cells and their differentiated derivatives underwent Affymetrix gene expression profiling as part of the Stem Cell Genomics Project. A set of 45 samples were profiled in biological triplicate and hybridized to the MOE430 GeneChip set. Mean intensity values for each biological triplicate are plotted in log scale on the Y-axis, with an approximate cutoff of 1000 demarcating detection status of each gene represented. Transcript expression levels of genes positively (*Nanog, Sox2*), negatively (*Tdrd7* and *Mef2a*), and not (*Myog*) correlated to *Oct4* are displayed. Detection calls of ‘Present’ for *Oct4* are depicted by solid black squares. ‘Absent’ calls are represented by open black squares.

As a result of this analysis 1299 probesets (1155 unique transcripts) were found to be correlated to *Oct4*. Seventy-five probesets (69 transcripts) were negatively correlated, while 1224 probesets (1086 transcripts) were positively correlated. The validity of this method for the identification of genes related to stem cell identity is assured by the presence of genes which have previously defined roles in ESCs such as *Utf1, Fgf4, Nanog*, and *Sox2* which were correlated to Oct4 in 100%, 99%, 97% and 49% of the trials respectively. Comparison of the transcript expression levels of *Oct4* and correlated *Nanog, Sox2, Tdrd7, Mef2a*, and uncorrelated *Myog* across all samples utilized in this analysis demonstrates the range of *Oct4* expression in these samples and also lends meaning at a biological level to the statistical analysis performed (Figure 1).

### GO Categorization of Oct4 Correlated Genes

In order to gain insight into the functions of *Oct4* correlated genes, GOstat analysis [24] was performed. As a result of this analysis a number of gene ontology (GO) categories were found to be correlated to Oct4 expression. Many over-represented terms were related to transcription and DNA replication (nucleic acid binding, DNA helicase, nucleolus), RNA processing (rRNA processing, splicesome complex, and RNA splicing), and cellular localization (nucleolus and Cajal body). Many under-represented terms were related to inter-cellular communication (cell communication, receptor activity, signal transduction). A complete output from GOstat is provided (Supplemental Table S3). Because this method of analysis is highly dependent upon the GO categories associated with a specific gene, the use of alternate GO databases can result in divergent findings. Moreover, such analyses are limited by the availability of databases which possess accurate annotations that keep pace with current research.

**Figure 2:**
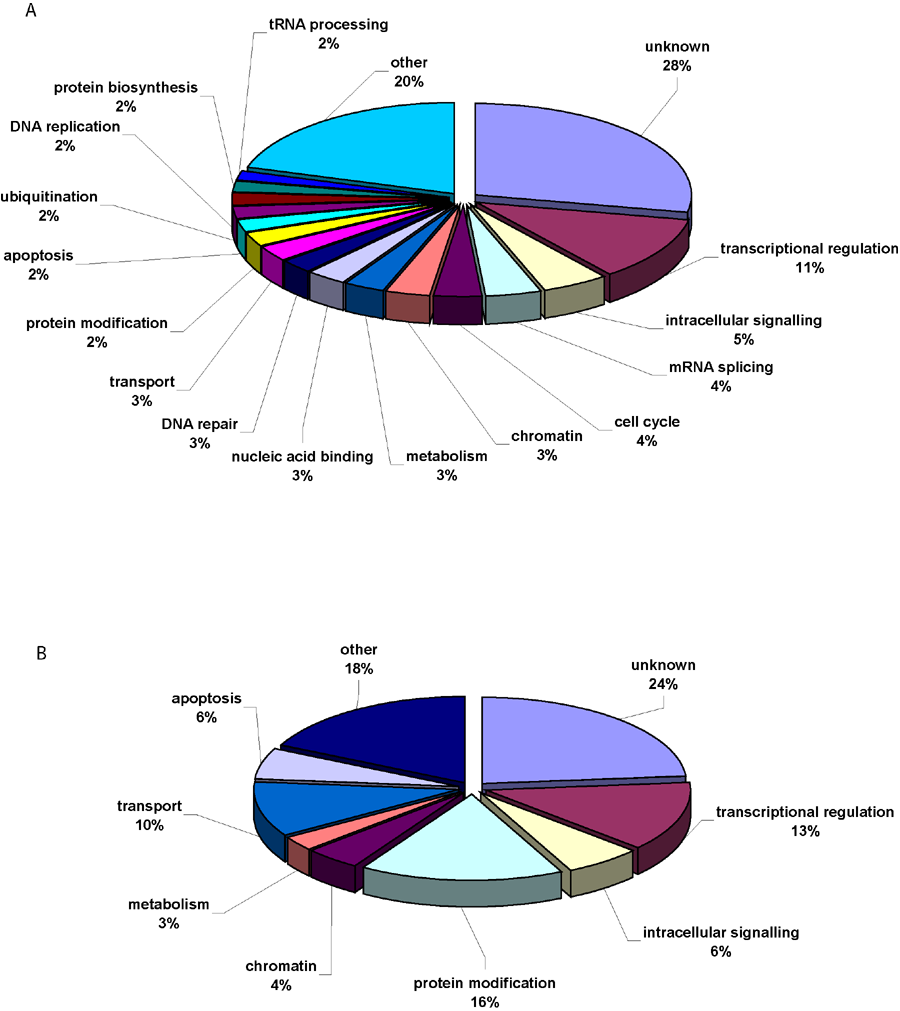
Functional classification of *Oct4* correlated genes. Manual curation of databases reveals highly enriched Gene Ontology (GO) categories for *Oct4* positively (A) and negatively (B) correlated genes. Numbers displayed represent percentage of unique transcripts attributed to each category with only the most abundant categories listed individually.

To overcome these limitations, further refinement of GO classifications for the *Oct4* correlated genes was performed by manual curation of a wide variety of databases such as *NetAffx, GeneCards, Ensembl, Stanford Source, Bioinformatics Harvester*, and *PubMed* (Supplemental Table S2). This analysis revealed that the categories transcriptional regulation, intracellular signaling, mRNA splicing, cell cycle, DNA repair, and chromatin were highly represented within the positively *Oct4* correlated genes. Categories highly represented within the negatively correlated genes included transcriptional regulation, protein modification, transport, intracellular signaling, and apoptosis. A summary of these findings is provided in Figure 2 with representative genes in highly enriched categories provided in Table 1. Of note, these findings are highly consistent with a previously published GO analysis performed following *Oct4* knockdown in hESC [25].

**Table 1:** Categories of Genes Identified as Oct4 Correlated.

Chromatin Structure	Nuclear Architecture	DNA Repair	Apoptosis	Cell Cycle Control
Arid1a	Pml	Blm	Aatf	Anapc10
Arid5b	Pum1	Brca1	Api5	Bub1
Ash1l	Coil	Chk1	Aven	Ccna2
Ash2l	Ncl	Ddb1	Bag4	Ccnb1
Cdyl	Mep50	Fancd2	Ciapin1	Ccnb2
Rest	Nup54	Lig1	Commd10	Ccne1
Jarid1b	Nup160	Lig3	Gtse1	Ccnf
Jarid2	Gemin4	Mre11a	Opa1	Chfr
Nasp	Gemin5	Msh2	Siva	Cdk5rap3
Phc1	Sfrs2	Parp1	Spinl	Cul2
Rnf134	Snrpn	Rad17	***Bin1***	D14Abb1e
Setdb1	Snrpa	Rad51	***Blp1***	Gstp1
Suz12	Snrpa1	Trp53	***Serpinb9***	Igf2bp1
***Bmi1***	Snurf	Xrcc5	***Sh3glb1***	Jarid1b
***Phc3***	Sf3b14	***Tdrd7***	***Casp6***	Nipp1

Highly represented Gene Ontology categories as identified by manual curation of databases such as *NetAFFX, GeneCards, Ensembl, Stanford Source*, and *Bioinformatics Harvester* and *PubMed.* Representative genes in each category are provided. Positively correlated genes are displayed in normal font. Negatively correlated genes are displayed in bold italics.

### Target Gene Validation

To validate our premise that this analysis would lead to the identification of Oct4 direct transcriptional targets, we performed a screen scanning the genomic region from 2 kb upstream of the transcriptional start site to 2 kb downstream from the 3-prime end of the transcribed region of the correlated genes for the presence of neighboring *Oct4* and dimerization partner *Sox2* binding sites (Supplemental Material and Methods). As a result of this analysis 392 genes were found to possess at least one putative composite binding site (Supplemental Table S4) with several genes such as *Oct1/Pou2f1, Smyd3*, and *Ranbp17* containing multiple (17, 16, and 14 respectively) putative sites, which may reflect a requirement for strict regulatory control of these genes throughout development. Although one might predict that genes containing multiple binding sites would show a higher degree of correlation to *Oct4*, a very cursory analysis of the data reveals that this is in fact not the case. Genes containing 1 *Oct4*/*Sox2* binding site (and % correlation) are: *Lig3* (+62), *Kctd3* (+91), *Bin1* (−41), *Bmi1* (−55), *Nasp* (99 and 79-two probesets). Genes containing from 5 to 10 sites include: *Insig2* (−61), *Ipo11* (+92), *Myst4* (+94), *Nr6a1* (52 and 53) and *Strbp* (52). Genes with greater than 10 sites are: *Pou2f1* (+50), *Ranbp17* (+99), and *Smyd3* (+45).

**Figure 3:**
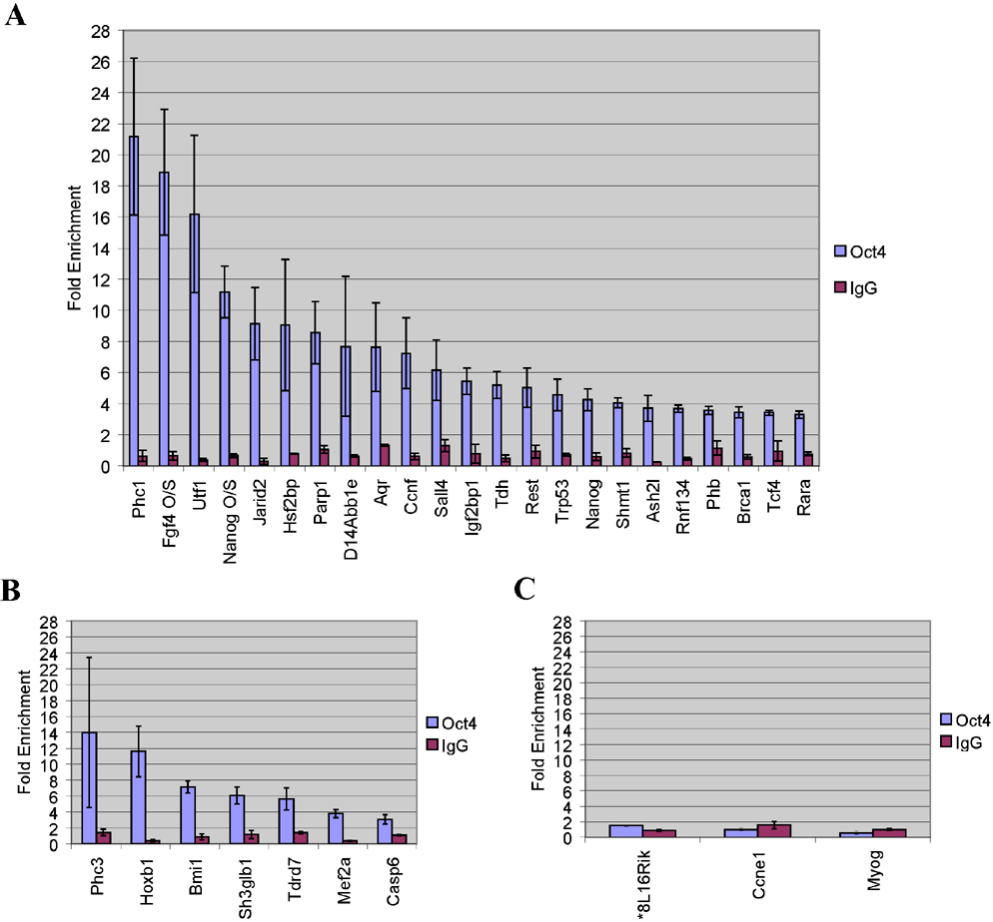
Validation of Oct4 targets. Chromatin immunoprecipitation (ChIP) assays were performed with *Oct4* and *IgG* antibody and no antibody as a negative control followed by Quantitative Real-time PCR analysis (ChIP/QRT-PCR) for putative positively regulated *Oct4* target (A), negatively regulated Oct4 target (B), and non-validated (C) genes. *8L16Rik represents *1110008L16Rik.* Results are from two independent ChIP assays, with duplicate QRT-PCR assessment for each. Error bars denote standard error of the mean (SEM).

Validation of 28 of these loci by chromatin immunoprecipitation (ChIP) followed by quantitative real-time PCR (QRT-PCR) confirmed the identification of 26 *Oct4* direct transcriptional targets (Figure 3; Supplemental Table S6). Notably, since the completion of our studies, these findings have been confirmed by several groups [11], [12], [25], [26], [27].

Further examination of these directly regulated target genes in the context of the correlated gene-list reveals important insights into how *Oct4* regulates pivotal pathways involved in controlling pluripotency, self-renewal and early lineage commitment.

### Oct4 Correlated Genes are Implicated in Chromatin Regulation

Recent experiments indicate that chromatin organization is dynamic and is subject to regulatory mechanisms that enforce the transcriptional potential of the genome during cellular commitment and differentiation. Chromatin is remodeled into transcriptionally permissive or repressive conformations by complexes that covalently modify histones, act in an ATP-dependent manner to reposition nucleosomes along DNA, or facilitate histone exchange. Several complexes have been identified including SWI/SNF, ISWI, INO80, and M1-2/CHD, and Trithorax group (TrxG), and Polycomb group (PcG) proteins which mediate chromatin remodeling by facilitating epigenetic modification of histone tails to activate or repress gene expression, respectively [28].

Thirty five genes implicated in chromatin remodeling were correlated to Oct4. Putative positive target genes include SWI/SNF members *Smarcc1*, AT rich interactive domain (Swi1 like) containing proteins (ARID domains) *Arid1a, Arid5b, Jarid1b* and *Jarid2*, which was confirmed as a direct *Oct4* target. Notably, these ARID domain containing proteins, a subset of the Jumonji C family, have recently been associated with histone demethylase activity [29]. Several other genes containing MYST, SET, and CHROMO, and BROMO domains, which facilitate or recognize specific histone modifications, were also identified.

*Rest* has been implicated in the repression of neuronal specific genes via its ability to recruit cofactors such as histone deacetylases (HDACs), Corest, Sin3, and Mecp2 [30]. The identification of *Rest* as a direct *Oct4* target, in light of its role in maintaining chromatin plasticity throughout neurogenesis, [31] provides a mechanistic understanding of *Oct4's* role in promoting neural differentiation [3]. Ironically, *Rest* has recently been described as both a tumor suppressor [32] and an oncogene [33]. The identification of *Corest* and *Mecp2* as respectively positively and negatively correlated to *Oct4* may provide insight into the dynamic nature of *Rest* co-repressor complexes throughout development that could explain these seeming incongruities. Furthermore, this hypothesis is supported by the recent description of the changing Rest-regulon in the progression from embryonic stem cells to neural stem cells (NSC) to differentiated neurons [34]

Importantly, several members of the TrxG and PcG of transcription factors such as *Ash1l, Suz12, Ash2l, Phc1*, and *Rnf134, Bmi1,* and *Phc3* were correlated to Oct4, with the five latter genes validated as *Oct4* targets. Diverse functions for PcG and TrxG genes in cancer, cell cycle control, and stem cell function have recently been described [35], [36], [37], [38]. The direct transcriptional regulation of several members of these complexes places Oct4 central to the coordination of these activities. The localization of Suz12, a member of Polycomb Repressor Complex 2 (PRC2) at many Oct4 repressed loci in ESC [37], [39] provides indication that Oct4-Polycomb interaction may play a significant role in the active repression of lineage. Furthermore, knock-down or overexpression of Oct4 has been shown to result in perturbed expression of several members of PcG and TrxG that we have identified as Oct4 targets and has led to loss of the pluripotent state [26], [27]. A comparison of the results of this study to the previous studies can be found in Table 2. Taken together, these data provide strong support for Oct4's role in maintaining chromatin structure in mESC via regulation of and interaction with a unique constellation of PcG and TrxG complexes.

**Table 2:** Cross-Study Comparison of Oct4 Target Genes

Gene Symbol	Campbell et al.	Loh et al. [12]	Boyer et al. [11]	Ivanova et al. [26]	Matoba et al. [27]
	ChIP-PCR in mESC	ChIP-PET in mESC	ChIP-ChIP in hESC	Perturbed expression following Oct4 shRNA	Perturbed Expression following manipulation of Oct4 expression (up or down)
**Phc1**	**✓**	**✓**	**-**	**✓**	**✓**
**Fgf4**	**✓**	**-**	**-**	**✓**	**✓**
**Utf1**	**✓**	**-**	**-**	**✓**	**✓**
**Nanog O/S**	**✓**	**✓**	**✓**	**-**	**✓**
**Jarid2**	**✓**	**-**	**✓**	**-**	**✓**
**Hsf2bp**	**✓**	**-**	**-**	**✓**	**✓**
**Parp1**	**✓**	**-**	**-**	**-**	**✓**
**D14Abb1e**	**✓**	**-**	**-**	**-**	**✓**
**Aqr**	**✓**	**-**	**-**	**-**	**-**
**Ccnf**	**✓**	**-**	**-**	**-**	**✓**
**Sall4**	**✓**	**-**	**-**	**-**	**✓**
**Igf2bp1**	**✓**	**-**	**-**	**-**	**✓**
**Tdh**	**✓**	**-**	**-**	**✓**	**✓**
**Rest**	**✓**	**✓**	**✓**	**✓**	**✓**
**Trp53**	**✓**	**-**	**-**	**-**	**✓**
**Nanog**	**✓**	**-**	**-**	**-**	**✓**
**Shmt1**	**✓**	**-**	**-**	**-**	**✓**
**Ash2l**	**✓**	**-**	**-**	**-**	**✓**
**Rnf134**	**✓**	**-**	**-**	**-**	**-**
**Phb**	**✓**	**-**	**-**	**-**	**✓**
**Brca1**	**✓**	**-**	**-**	**-**	**✓**
**Tcf4**	**✓**	**-**	**✓**	**-**	**✓**
**Rara**	**✓**	**-**	**-**	**✓**	**✓**
**Phc3**	**✓**	**-**	**-**	**-**	**✓**
**Hoxb1**	**✓**	**✓**	**✓**	**-**	**✓**
**Bmi1**	**✓**	**-**	**-**	**-**	**✓**
**Sh3glb1**	**✓**	**-**	**-**	**✓**	**-**
**Tdrd7**	**✓**	**-**	**-**	**-**	**-**
**Mef2a**	**✓**	**-**	**-**	**-**	**-**
**Casp6**	**✓**	**-**	**-**	**✓**	**✓**

Comparison of validated Oct4 targets to previous studies employing ChIP-Pet, ChIP-ChIP and expression analysis following Oct4 knockdown or overexpression. Discordant findings in the ChIP based approaches may be explained by the use of promoter based chips or stringency of analysis. Although shRNA knockdown of Oct4 reveals few genes that are predicted to be bona fide Oct4 targets that are identified in common, comparison to the dataset in Matoba et el. [27] reveals that expression of most of the targets identified in this study are in fact perturbed upon up or downregulation of Oct4. Discordant findings between this study and Matoba et al. may be impacted by the temporal nature of Oct4 regulation of these target genes as has been described previously for the Rest regulon (Sun et al. [34]).

The negative correlation between *Bmi1* and *Oct4* was surprising in light of its role in maintaining hematopoietic and neuronal stem cells (HSCs, NSCs). Although necessary for self-renewal of HSCs and NSCs, expression of *Bmi1,* which leads to chromatin condensation and stable gene silencing [40] may be inconsistent with self-renewal in pluripotent cells. Pluripotency involves the ability to repress genes whose expression would result in a loss of potential while retaining the ability to reawaken these transcriptional programs upon differentiation. Therefore, while transcriptional repression is necessary in both pluripotent cells and their differentiated progeny, the means to accomplish it may, of necessity, be entirely different.

PcGs exist as developmentally regulated multi-subunit complexes [41]. Therefore it is predicted that alterations in the balance of PcG members would have profound implications for maintenance of the stem cell state. If, as anticipated above, inappropriate upregulation of *Bmi1* (and/or *Phc3*) leads to the repression of genes that are required for pluripotency, this may ultimately be manifested in a cell's inability to differentiate and may provide a partial explanation for the oncogenic roles of these proteins. Conversely, it is postulated that downregulation of other PcG members such as *Phc1* would result in the de-repression of genes required for differentiation which would compromise self-renewal [42].

### Cell Cycle Control in Stem Cells Requires Inactivation of pRb for Self-Renewal, Activation for Differentiation

Carefully regulated execution of cell cycle progression is accomplished in stem cells by a unique constellation of genes which impact self-renewal and lineage commitment. Activation of intracellular signaling pathways such as *PI3K, Ras/Raf*, and *Jak*/*Stat* by molecular cues emanating from the stem cell niche mediate phosphorylation events which control the activity of cyclin/CDK complexes and culminate in the modulation of genes (such as *pRb* and *Trp53*) that are implicated in cell cycle checkpoint, cell cycle exit, and differentiation [43].

Assessment of GO terms revealed that 38 cell-cycle related genes were positively correlated to *Oct4* including *Cdc25a, Gspt1, Ppp1r8, Ccnb2, Ccne1, Ccna2*, Ccnb1, and *Ccnf*. Validated *Oct4* target *Ccnf* is implicated in cell cycle control at the G1/S and G2/M checkpoints and has recently been associated with the maintenance of *pRb* in a hyperphosphorylated, inactive state [44]. The role of *Ccnf* in this process may in part be due to the E3 ubiquitin ligase domain of *Ccnf* to mediate the degradation of phosphatases such as *Pp1* involved in the sequential activation of *pRb* through G1/S and G2/M [45] *.* Conversely, the significantly Oct4 correlated (92%) Pp1 negative regulatory subunit Nipp1 (Ppp1r8) may facilitate the functional inactivation of *pRb.* This hypothesis is consistent with the requirement of *Nipp1* in early embryonic development [46] and points toward a potential role for *Nipp1* in tumorigenesis [47]. In addition to its role in cell cycle control is also involved in mRNA splicing, and transcriptional repression through interactions with the PcG complexes making it an important putative *Oct4* target, capable of integrating the diverse functions of cell cycle control, alternate splicing, chromatin structure, and transcriptional regulation [46].

Based upon this analysis it is predicted that alterations in the expression of Oct4 correlated genes such as *Ccnf* or *Nipp1* that impact the functional status of *pRb* (or *pRb* family member *p107*) would have profound consequences. Inactivation of *pRb* is required for self-renewal; activation of *pRb* is obligatory for cell cycle exit and differentiation. An imbalance in either of these processes, possibly emanating from deregulated signaling from the stem cell niche or mutations in the key regulators would lead to unrestrained cellular proliferation.

### Genes Involved in Apoptosis and DNA Repair are Correlated to Oct4 and are Implicated in Stem Cell Differentiation

Prevailing thought holds that the initial stages of apoptosis involve the caspase mediated induction of DNA strand breaks and either the recruitment of DNA repair genes that act in concert to halt cell cycle progression and restore genomic stability or, if the damage is not able to be repaired, in further cleavage of DNA, nuclear blebbing, and other processes which have been elegantly and thoroughly described elsewhere that culminate in programmed cell death. Both apoptosis and DNA repair are regulated by several multi-component complexes with the roles of *Trp53, Brca1*, and *pRb* being central to their coordination [48].

Analysis of the *Oct4* gene list revealed an important emerging theme; mechanisms to actively repress apoptotic pathways are involved in maintaining the stem cell state. Twenty-five apoptotic genes were positively correlated to *Oct4*, the majority of which, including *Aatf, Api5, Aven, Bag4, Commd10, Nipa*, and *Opa1,* function to inhibit apoptosis. In addition, *Bin1, Blp1, Serpinb9, Sh3glb1*, and *Casp6*, all apoptosis inducing genes, were found to be negatively correlated to Oc*t4*, with *Sh3glb1* and *Casp6* confirmed as targets.

Thirty genes implicated in DNA damage and repair, were positively correlated to *Oct4*. Members of the *Brca1* associated surveillance complex (BASC) including *Brca1, Msh2, Mre11a, Rad51, Blm, Chek1*, as well as *Parp1, Trp53, Fancd2, Tdrd7, and Xrcc5*, were included. The validation of *Trp53, Tdrd7, Brca1*, and *Parp1* as direct *Oct4* targets strengthens the importance of this group of genes in stem cell function.

The high frequency at which apoptotic genes were negatively correlated to *Oct4* and anti-apoptotic genes were positively correlated to *Oct4* implies that ‘anti-apoptosis’ is an important theme for maintaining the stem cell state. Conversely, this may also suggest that genes which modulate the initial response to aberrant chromatin structure, apoptosis, and DNA repair, may play important roles in lineage commitment. This notion is consistent with the role of tudor domain containing proteins (such as *Tdrd7*) in DNA damage response [49], *Casp3* in skeletal muscle differentiation [50], and the roles of *Parp1* [51], *Trp53* [52], and *Brca1* [53] to modulate differentiation. Interestingly, a relationship between DNA damage repair, chromatin remodeling [54], and histone deacetylation [55], all previously implicated in cellular differentiation, has recently been described. Moreover, knowledge of the normal developmental functions of these genes in cellular differentiation provides mechanistic insight into how these genes, when mutated, lead to cancer.

### Nuclear Architecture in Stem Cells Reinforces Their Defining Characteristics

The nucleus is the site of many processes that profoundly impact cellular phenotype including transcription, mRNA splicing, and DNA replication and repair. Research has revealed that in fact control of these activities is coordinated in a dynamic, spatio-temporal manner. The presence of specific nuclear structures (nuclear bodies; NBs), whose function is to concentrate key regulatory molecules, mainly to loci of actively transcribed genes, facilitates this coordination [56].

As a result of this analysis several key molecules whose presence is indicative of NBs were observed. *Pml* and *Coil* (Cajal Bodies and PML Bodies), *Gemin4* and *Gemin5* (Gems), *Nup35, 43, 54, 98, 133, 160, 188* (Nuclear Pore Complex), *Ncl* and *Nolc1* (Nucleolus) and 46 genes implicated in RNA metabolism (Splicing Speckles, Spliceosome, Exosome, and Cajal Bodies) were positively correlated to Oct4. Several genes implicated in nuclear transport such as *Ipo11, Kpna1, Tnpo2* and *3, Xpot, Gle1l, Xpo5* and *6* and direct Oct4 targets *Igf2bp1* and *Phb* were also positively correlated.

The incidence of nuclear bodies is incremental with cellular proliferative capacity and their localization is predominately to transcriptionally active regions of chromatin, although the mechanisms that direct their localization are largely unknown. Based upon the high degree of correlation of *Oct4* to constituents of NBs, it is conceivable that *Oct4* target binding may function to modulate the accessibility of local chromatin to these structures and thereby enforce the transcriptional potential of specific genetic loci in early development. The identification of Hoxb1 as a negatively regulated *Oct4* target is consistent with this hypothesis in light of the recent finding that in ESCs *Hoxb1*, although not expressed, is poised at the surface of its chromosome territory. In the initial stages of differentiation *Hoxb1* is transcriptionally activated which results in chromatin decondensation and reorientation of this locus to the nuclear centre [57]. Together, these findings lead us to predict that *Oct4* binding functions not only in the transcriptional repression of genes that would otherwise facilitate lineage commitment, but also presents a means whereby these loci are organized spatially within the nucleus so as to be poised for activation given the appropriate cue.

In addition to the normal physiological roles for NBs described above, they also play key roles in the response to DNA damage, DNA repair, apoptosis, and senescence. Loss of regulation in the recruitment and coordination of key genes contained in these structures (*Trp53, Pml, Brca1, Blm*, etc.) would be predicted to have profound implications in the ability of a cell to respond to signals that would lead to differentiation. Such dysregulation is associated with the accumulation of NBs at sites of DNA damage (DNA damage induced foci) and is implicated in several types of cancer such as acute promyelocytic leukemia (*Pml-Rara* translocation) and Bloom's Syndrome [56].

### Conclusions

**Figure 4:**
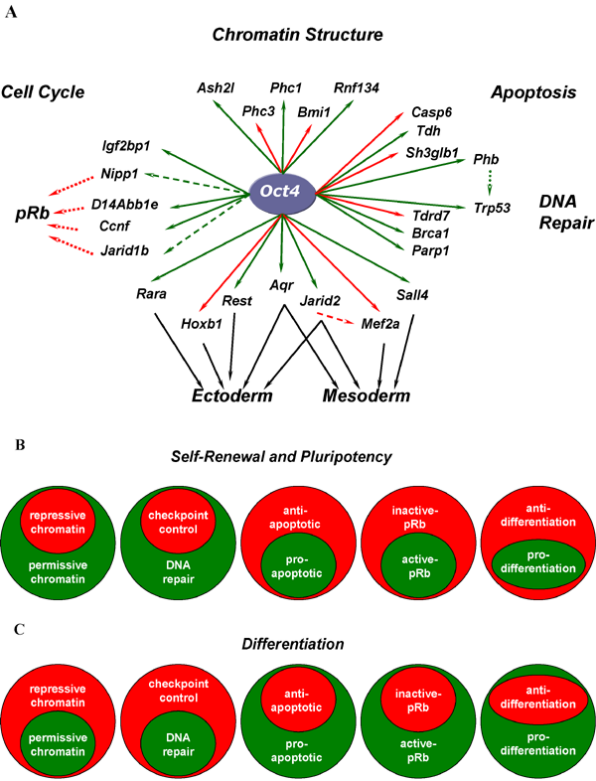
The *Oct4* transcriptional regulatory network. Validated Oct4 targets (A) are indicated by solid red or green lines. Red and green indicate negative and positive regulation, respectively for all cases. Dashed lines emanating from Oct4 indicate putatively regulated genes. Solid black lines represent potential regulatory nodes that could facilitate the directed differentiation of ESCs. The pressures that preserve stem cell function and modulate early lineage commitment are diametrically opposed. While Oct4 acts to maintain self-renewal and pluripotency in the undifferentiated ‘ES’ state by its modulation of genes that act to maintain permissive chromatin structure, DNA repair, anti-apoptosis, and inactive *pRb* (B), in differentiation the balance of these forces is altered to favour repressive chromatin structure, DNA checkpoint control, apoptosis, and active pRB which facilitate cellular commitment (C).

Through the use of gene expression data compiled from a vast collection of adult and embryonic stem cells and their differentiated derivatives we have performed a robust statistical analytic method to identify genes that are correlated to *Oct4*. Although several previous studies have mapped transcriptional targets of *Oct4*, we believe that this study provides further insight into the transcriptional regulatory networks, factors, and cofactors that modulate stem cell function. Importantly, our experiments have revealed hitherto unappreciated roles for *Oct4* for firstly, regulating chromatin structure in a state consistent with self-renewal and pluripotency, and secondly, facilitating the expression of genes that keeps the cell poised to respond to cues that lead to differentiation. Furthermore, our analyses has led to the elucidation of themes that are essential for maintaining ‘ES’ including permissive chromatin structure, nuclear architecture, cell cycle control, apoptosis, and DNA repair. Finally, we have identified 26 direct Oct4 transcriptional targets which may represent candidate regulatory nodes by which cell fate decisions could be directed to facilitate the use of hESCs in therapeutic and regenerative medicine (Figure 4A and Table S2).

### The Oct4 Transcriptional Regulatory Network

The expression of *Oct4* in various forms of human cancer [8], [9] and a recently described role for *Oct4* in adult stem cells [10] and the expansion of epithelial progenitor cells [7] supports the theory that cancer is a disease of stem cells. This theory postulates that cancers arise in stem cells or early committed progenitors [58] due to their inability to differentiate in a regulated fashion. *Oct4* directly regulates the transcription of genes such as *Trp53, Brca1, Parp1*, and *Bmi1* which play a central role in a cell's proclivity to undergo transformation, apoptosis, senescence, and now differentiation.

The process of development and the commitment to differentiate is guided by the ordered expression and repression of genes required to enforce specific transcriptional programs. Knowledge of the emerging Oct4 transcriptional regulatory network provides a means whereby we can begin to understand the molecular mechanisms that guide these processes and gain insight into aberrations that lead to disease. While the stem cell state is guarded by highly dynamic, complex, and interrelated mechanisms which impact the repertoire, location, and functional state of expressed genes, lineage commitment can be described as a process whereby the unlimited ability for self-renewal and potency are gradually restricted as a cell progresses from one steady state of gene expression to the next. These diametrically opposed states are mediated by a contrasted balance of forces that impact chromatin structure, nuclear architecture, cell cycle, DNA repair, and apoptosis (Figure 4B and C). Further examination of the interactions among the genes identified as a result of this study will provide a more thorough understanding of the pressures that guide cell fate. Critically, only by understanding the normal developmental function of a gene can we begin to understand the role that it may play in disease. Importantly, our experiments have defined how *Oct4*, as the master regulator of embryonic stem cell function, plays a central role in regulating key genes in pivotal pathways involved in controlling pluripotency, self-renewal and differentiation.

## Materials and Methods

### Stem Cell Culture and Isolation

The samples included in this study were obtained from various members of the Stem Cell Network in support of The Stem Cell Genomics Project. Full descriptions of the origin and experimental conditions used to derive each sample can be obtained from StemBase; **(** **http://www.scgp.ca:8080/StemBase** **).**

### Target Labeling and Hybridization

Total RNA (10 ug or 10–50 ng) was labeled as per manufacturer's suggested methods (Afymetrix, Santa Clara, California, USA). Briefly, following first strand and second strand cDNA synthesis, samples underwent a single round (10 ug starting material) or two rounds (10–50 ng starting material) of linear amplification using a T7 based *in vitro* transcription (IVT) kit (MegascriptT7, Ambion). During the final round of IVT, biotinylated nucleotides were incorporated into the nascent strand (Enzo Biotech, Farmington, Connecticut, USA) to produce the labeled target cRNA. Ten micrograms of cRNA were fragmented to reduce complexity and hybridized overnight to the MOE 430 GeneChip Set, according to standard protocol. The GeneChips were then washed and stained with Streptavidin R-Phycoerythrin (SAPE). Signal amplification was accomplished by subsequent staining with biotinylated anti-streptavidin, followed by an additional incubation with SAPE. Scanning and absolute analysis was performed in MAS 5.0 to generate the experiment (.exp), raw image (.dat), intensity (.cel) and absolute analysis (.chp) files. All samples were scaled to a target intensity of 1500 during analysis.

### Correlation Analysis

Normalized expression values for each probeset were obtained from MAS 5.0 (http://www.affymetrix.com/products/software/specific/mas.affx) and the mean expression value for each set of biological triplicates was calculated. The data were scaled by normalizing to the trimmed mean for all probesets in the chips (98%). Probesets that had a consensus detection call of present (P) in more than 7% and less than 93% of the samples were included in the analysis. The standard Pearson correlation coefficient (rho) between every probeset which passed the filter, to the *Oct4* probeset (1417945_at) was computed. A probeset is considered correlated to *Oct4* if the absolute value of rho is greater than or equal to 0.75. This computation was repeated 10,000 times with random subsets consisting of 65% to 70% of the data. Probesets that were correlated in at least 40% of the trials were retained for further analysis.

### GOStat Analysis

GOstat (http://gostat.wehi.edu.au/) was used to examine selected sets of probesets for over- and under-representation of GO terms, using MGI (http://www.informatics.jax.org/mgihome/) as GO to gene association database, and using false discovery rate correction. This method is sensitive to the GO annotations attached to the genes related to the probes, thus the result might change if another database (e.g. GOA) is used.

### Binding Site Analysis

The genomic region from 2 kb upstream of the transcriptional start site to 2 kb downstream from the 3-prime end of the transcribed region of the correlated genes was scanned for the presence of neighboring *Oct4* (ATGCAAAT) and *Sox2* (AACAAAG) binding sites. Global analysis of the *Oct4* correlated gene-list was performed in a conservative fashion based upon POU/HMG/DNA ternary complex assembly as determined by crystal structure assessment of *Fgf4* and *Utf1* [59]. First, the two components of the *Oct4* binding site, namely the POU specific domain (POU_S_) and the POU homeodomain (POU_H_) were forced to be consecutive in the sequence while independently in any direction, and in any of the two strands. A perfect match was required for POU_S_ (ATGC), and one mismatch was allowed at any of the four positions of POU_H_ (AAAT). Second, we defined the *Sox2* binding site as either AACAAAG, which corresponds to the predominant pattern, or the observed variations AACAAAT, or AACAATG, in any direction or strand. The maximum distance between *Oct4* and *Sox2* binding was constrained to 3 nucleotides.

Manual assessment of binding sites for a subset of the *Oct4* correlated genes as well as developmentally important regulators *Hoxb1* and *Tcf4* was performed in a less restrictive fashion. POU_S_ was held invariant while the POU_H_ (AAAT) was allowed to vary by one mismatch in any of the four nucleotide positions. Target sequence identification for the two POU domains relative to each other and to the *Sox2* site were not restricted in order, orientation, or strand. Finally, as has been observed for Oc*t*4/*Sox2* cooperative binding on *Opn* [60], the distances between the *Oct4* and *Sox2* binding sites was relaxed and allowed to span up to 100 nucleotides.

### Chromatin Immunoprecipitation

Chromatin Immunoprecipitation (ChIP) assays were performed using the Chromatin Immunoprecipitation Assay Kit (Upstate Biotechnology, Lake Placid, NJ, USA). Briefly, 5×10^6^ J1 ESCs were cross-linked with 1% formaldehyde for 15 minutes at room temperature. Cells were washed three times in ice-cold PBS with protease inhibitors and lysed in buffer provided to which protease inhibitors were also added. The cells were sonicated to an average size of 1500 bp and 250 ug of input chromatin was used for each assay. Immunoprecipitation was performed overnight at 4°C with *Oct4* antibody (Santa Cruz Biotechnology, Inc., Santa Cruz, California) and no antibody as a negative control.

### Quantitative Real-time PCR

Quantitative PCR was performed using primers that flanked the regions containing putative *Oct4* and *Sox2* binding sites with the MX4000 (Stratagene, La Jolla, California, USA) using iQ SYBR Green Supermix (BioRad, Hercules, California.). The following cycling parameters were employed: 96° 10 minutes, followed by 40 cycles of 96°C for 30 seconds, 57°C for 1 minute, and 72°C for 45 seconds. Primer sequences for each amplicon are described in Supplemental Table S2. Each result represents two independent ChIP assays with duplicate QRT-PCR analyses performed on each target gene for each assay. 100% amplification efficiency is assumed based on ΔΔC_t_ values of ∼3.3 between each point of a 10-fold serial dilution curve performed for a subset of the amplicons. A 2-fold enrichment therefore represents the minimum threshold for confirmation as an *Oct4* target. Error bars denotes the Standard Error of the Mean. Subsequent to QRT-PCR analysis, each amplicon underwent DNA sequence analysis on and ABI 3730 to confirm identity.

## Supporting Information

Table S1 Samples used for Oct4 correlation analysis
(0.08 MB DOC)

Table S2 Summary of Oct4 Correlated Genes with Probeset ID, gene symbol, gene name, direction and percentage of correlation, chromosomal location, summary GO category used for Figure 2 and GO biological process were listed when known.
(0.28 MB XLS)

Table S3 GoStat Analysis
(0.33 MB XLS)

Table S4 Oct4/Sox2 putative binding site analysis with Gene symbol, RefSeq or Ensembl ID, putative binding sequence, and location in transcript enumerated
(0.11 MB XLS)

Table S5 Primer sequences for Oct4 target validation by ChIP/QRT-PCR
(0.06 MB DOC)

Table S6 Annotation of Oct4 targets.
(0.09 MB DOC)
