# Supplementary material for: Oct4 Targets Regulatory Nodes to Modulate Stem Cell Function
Source: PLoS One. 2007 Jun 20;2(6):e553. doi: 10.1371/journal.pone.0000553 (PMC1891092; doi:10.1371/journal.pone.0000553)
Supplement: Table S1 — Samples used for Oct4 correlation analysis (0.08 MB DOC) [file pone.0000553.s001.doc]

| **Table S1.** Samples used for Oct4 Bootstrapping Analysis | | | |
| --- | --- | --- | --- |
| **Sample ID** | **Sample Name** | **Sample ID** | **Sample Name** |
| ***128*** | **J1 ES** | ***271*** | Neurospheres |
| ***242*** | **J1 ES** | ***198*** | Neurospheres |
| ***249*** | **J1 14D EB** | ***272*** | Neurospheres P107-/- |
| ***206*** | **R1 ES** | ***255*** | Mammospheres |
| ***217*** | **R1 14D EB** | ***256*** | Mammospheres, 6D Differentiation |
| ***153*** | **V6.5 ES** | ***269*** | Myospheres |
| ***175*** | **V6.5 14D EB** | ***274*** | Myospheres Sca1+ |
| ***169*** | **D4 ES** | ***270*** | Myospheres 7D Differentiation |
| ***167*** | **D4 ES Attached** | ***199*** | Adipose Spheres |
| ***168*** | **D4 ES Detached** | ***200*** | Dermis Spheres |
| ***166*** | **C2 ES** | ***147*** | BM Sca1+CD45+ |
| ***164*** | **C2 ES Attached** | ***233*** | BM Sca1-CD45- |
| ***165*** | **C2 ES Detached** | ***234*** | BM Sca1-CD45- |
| ***219*** | **R1 Oct4 GFP Serum 6999** | ***235*** | BM Sca1-CD45- |
| ***220*** | **R1 Oct4 GFP Serum 6473** | ***294*** | BM Lin-Sca1+cKit+ |
| ***132*** | **P19 EC 2D Aggregated** | ***295*** | BM Lin-Sca1-cKit+ |
| ***129*** | **P19 EC 1D Monolayer** | ***296*** | BM Lin-Sca1-cKit- |
| ***130*** | **P19 EC 1D Aggregated** | ***291*** | BM Lin-Sca1+cKit- |
| ***131*** | **P19 EC 2D Monloayer** | ***293*** | BM Lin- |
| ***196*** | D3 30D Osteoblast Differentiation | ***292*** | BM Total Population |
| ***240*** | Retinal Spheres Primary | ***236*** | BM Mast Cell Precursors |
| ***232*** | Retinal Spheres First Passage | ***237*** | BM Mature Mast Cells |
|  |  | ***137*** | C2C12 Myoblasts |
