# Supplementary material for: Oct4 Targets Regulatory Nodes to Modulate Stem Cell Function
Source: PLoS One. 2007 Jun 20;2(6):e553. doi: 10.1371/journal.pone.0000553 (PMC1891092; doi:10.1371/journal.pone.0000553)
Supplement: Table S5 — Primer sequences for Oct4 target validation by ChIP/QRT-PCR (0.06 MB DOC) [file pone.0000553.s005.doc]

| **Table S5.** Primer Sequences for Oct4 Target Validation by ChIP/QRT-PCR | | |
| --- | --- | --- |
| **Target** | **Forward Primer** | **Reverse Primer** |
| ***Aqr*** | 5’-TTCAGAGGACTCCACCACAGGA-3’ | 5’-CGTTGGGATGGCTACCTTTCTC-3’ |
| ***Ash2l*** | 5’-TGGGGAGGACTGTGGAAGGACT-3’ | 5’-TGGAACACAGCCACTGTGCTCT-3’ |
| ***Bmi1*** | 5’-ACCTAAATTTGTACAGTCCC-3’ | 5’-CATTGGTAACTTTTGCTTC-3’ |
| ***Brca1*** | 5’-ATGCACTGAACTCCAAGCAGGT-3’ | 5’-CAGCCTAGGCTGTGAAGCAAGA-3’ |
| ***Casp6*** | 5’-GATCCAGCCGAGCAGTACAAGA-3’ | 5’-CCTGTGAAAGGGCTTAGCAGTG-3’ |
| ***Ccnf*** | 5’-GGGGAACCCTCACACTGTCTCT-3’ | 5’-CTTCCAGAGGCTACCAAAAGCA-3’ |
| ***D14Abb1e*** | 5’-AAGCTGATGGTGGCCTCCTAA-3’ | 5’-CCTACGTAAGCTAATGCT-3’ |
| ***Hoxb1*** | 5’-CCCTCCCTCTGGTCCCTTCTTT-3’ | 5’-GGGCACATGTGATCTCTC-3’ |
| ***Hsf2bp*** | 5’-ACTGGGCGTCAGGACAGAATG-3’ | 5’-TCCCATGTGCCCATAGTTTCC-3’ |
| ***Igf2bp1*** | 5’-GGCCCCTAGATCTTGAATGAGG-3’ | 5’-CAGCACGTGGAAAACACCAAAA-3’ |
| ***Jarid2*** | 5’-TGTTTGGTTTCATTTCCC-3’ | 5’-CTATAATGTGCCCCACAA-3’ |
| ***Mef2a*** | 5’-TGAAGACCCAGAACCCATACCC-3’ | 5’-GAGGCTCTTCCCCACTTTCTTC-3’ |
| ***Nanog*** | 5’-TGTTTTAGTGTGGGTATGGGCC-3’ | 5’-TGTGGTCCCTCCTCTTTC-3’ |
| ***Parp1*** | 5’-CTGAAGCTGTCATCTTGGTGCC-3’ | 5’-GGCATTCTGCCTAGCACTCAGT-3’ |
| ***Phb*** | 5’-ATGCACTATCCATTGCGCCA-3’ | 5’-GGGATTAAAGCGTCCGCCA-3’ |
| ***Phc1*** | 5’-ACCTCAGCCATAATTAGTTG-3’ | 5’-AAACCCTTTCACCTCTCC-3’ |
| ***Phc3*** | 5’-CGTATCCATTTATGTATGTGCT-3’ | 5’-TTACAAAAAGCCAATGGTGTA-3’ |
| ***Rara*** | 5’-GTCAACAGAGGACACAGTGGCC-3’ | 5’-TGCAATCCCTCTGCCTCAGC-3’ |
| ***Rest*** | 5’-TGGTTGGAAATTCTGCTCTG-3’ | 5’-TCCTGGAAGTCACTGGGATT-3’ |
| ***Rnf134*** | 5’-TATGAGTGCTGTTTCCATA-3’ | 5’-CTTGTGGTTTCTCCTTTG-3’ |
| ***Sall4*** | 5’-TAAGGGATCCTGTCTGGCACAC-3’ | 5’-TTGCAAGCTCTGGGAAATGACT-3’ |
| ***Sh3glb1*** | 5’-TCTAATCAGACTCATTTATGGG-3’ | 5’-CCGCAGCAATCTAAATCT-3’ |
| ***Shmt1*** | 5’-ATCAGAGCCACCTGGAAACAGA-3’ | 5’-GCTGCTGGATGCTTAAAGAAGC-3’ |
| ***Tcf4*** | 5’-atgacaatccagagagcagaag-3’ | 5’-GCTCGATGACTCTATCCGTGTAA-3’ |
| ***Tdh*** | 5’-GCAGTTATTCTTCCTCCAGCGG-3’ | 5’-GCTTGAGGGGAAGAGTGCATTT-3’ |
| ***Tdrd7*** | 5’-AGAGGGAGTGCTTCCGTTTTCA-3’ | 5’-GCCATTAAAGGCTGCTCACAAC-3’ |
| ***Trp53*** | 5’-GCAACTTCTAGAAACCCTGGGG-3’ | 5’-TTGGGAAATGGAGGCCTGG-3’ |
| ***Myog*** | 5’-CCGTCCGTCCAAGACAACCC-3’ | 5’-CCCCCCTCTAAGCTGTTGC-3’ |
| ***Ccne1*** | 5’-TCGCCTGCAGGAGAGGATGA-3’ | 5’-CGTGGACCCCTGCTCTTTCATC-3’ |
| ***1110008L16Rik*** | 5’-CATGCAGGGAAATGGAGCC-3’ | 5’-TCACTTCCCTGCAGCTGTCC-3’ |
| ***Utf1*** | 5’-CTCAGTTTGAATACTCTGGGCC-3’ | 5’-GAGGACCTTGGAATTTCAGCT-3’ |
| ***Fgf4 [1]*** | 5’-AGACTTCTGAGCAACCTCCCGAA-3’ | 5’-CAACTGTCTTCTCCCCAACACTCT-3’ |
| ***Nanog [1]*** | 5’-GTCTTTAGATCAGAGGATGCCCC-3 | 5’-CTACCCACCCCCTATTCTCCCA-3’ |

1. Kuroda T, Tada M, Kubota H, Kimura H, Hatano S-y, et al. (2005) Octamer and Sox Elements Are Required for Transcriptional cis Regulation of Nanog Gene Expression. Mol Cell Biol 25: 2475-2485.
