# Supplementary material for: Oct4 Targets Regulatory Nodes to Modulate Stem Cell Function
Source: PLoS One. 2007 Jun 20;2(6):e553. doi: 10.1371/journal.pone.0000553 (PMC1891092; doi:10.1371/journal.pone.0000553)
Supplement: Table S6 — Annotation of Oct4 targets. (0.09 MB DOC) [file pone.0000553.s006.doc]

| **Table S6.** Annotation of Oct4 Targets | | | |
| --- | --- | --- | --- |
| **Target** | **Correlation to Oct4** | **Function** | **Potential or Known Role in Stem Cells, Development and/or Disease** |
| ***Aqr*** | +90% | RNA dependent RNA polymerase | Expressed in mesoderm, neural crest, and neuroepithelium. Gene trap insertion mutants (not null mutants however) are normal in viability and fertility [1]. Role for *Aqr* in RNAi in mammalian oocytes discounted [2]. |
| ***Ash2l*** | +99% | Transcriptional Activation | Trithorax group protein downregulated in megakaryocyte but not in erythroid differentiation. Highly expressed in leukemic cell lines [3]. |
| ***Bmi1*** | -55% | Transcriptional Repression | Component of Polycomb Repressive Complex 1 (PRC1)/Maintenance complex implicated in self-renewal of neural [4] and hematopoietic [5] stem cells. |
| ***Brca1*** | +69% | DNA Damage Response | Part of the BASC Complex responsible for cell cycle checkpoint in response to DNA strand breaks. Mutations in Brca1 are responsible for at least 80% of inherited breast and ovarian cancers (BOC) [6]. |
| ***Casp6*** | -69% | Induction of Apoptosis | Effector Caspase, cleaves *Parp1* in vitro. Loss of expression in gastric cancer [7]. |
| ***Ccnf*** | +100% | Regulation of Cell Cycle | Homozygous mutants are embryonic lethal, MEF mutants display cell cycle defects with impaired cell cycle reentry from quiescence [8]. |
| ***D14Abb1e*** | +100% | Unknown | *Retinblastoma-associated protein 140 (Rap140)* Tumor Antigen expressed in Cutaneuos T-cell Lymphoma, Leukemia, and Melanoma [9]. |
| ***Hsf2bp*** | +100% | Transcriptional Regulation | Modulates transcriptional activity of *Heat Shock Factor 2* in testis [10]. |
| ***Hoxb1*** | --- | Transcriptional Regulation | Developmentally important homeobox transcription factor. Implicated in neuronal development [11]. |
| ***Igf2bp1*** | +99% | Nucleic Acid Binding | High expression in embryonic development and in *CD34*+ cord blood samples. Re-expression in 5’-azacytidine treated adult *CD34*+ BM samples. Expressed in breast cancer [12]. |
| ***Jarid2*** | +100% | Chromatin Remodelling | Transcriptional repressor required for neural tube formation and normal heart development [13]. |
| ***Mef2a*** | -49% | Transcriptional Regulation | Activates transcription of muscle specific genes. Mutations in *Mef2a* associated with Coronary artery disease [14]. |
| ***Parp1*** | +100% | DNA Repair | Mediates *Nad*+ dependent transcriptional repression of chromatin [15]. |
| ***Phb*** | +98% | DNA Metabolism | Induces transcriptional activation of *Trp53*. Mutations in Phb associated with sporadic breast cancer [16,17]. |
| ***Phc1*** | +100% | Transcriptional Repression | Component of Polycomb Repressive Complex 1 (PRC1)/Maintenance complex. Implicated in self-renewal of hematopoietic stem cells [18] and cardiac morphogenesis [19]. |
| ***Phc3*** | -40% | Transcriptional Repression | Component of Polycomb Repressive Complex 1 (PRC1)/Maintenance complex. |
| ***Rara*** | +49% | Transcriptional Regulation | Responsive to morphogen Retinoic Acid. Controls cell function by direct regulation of gene expression. Implicated in Acute Promyelocytic Leukemia due to translocation with PML [20]. |
| ***Rest*** | +76% | Transcriptional Repression | Maintains neural stem cells in undifferentiated state [21]. Modulates chromatin plasticity of neuronal precurcors [21]. Putative tumor suppressor in mammary epithelia [22]. |
| ***Rnf134*** | +100% | Transcriptional Repression | Polycomb group protein, interacts with *Rnf2* [23]. |
| ***Sall4*** | +99% | Transcriptional Regulation | Spalt transcription factor implicated in Okihiro Syndrome which is phenotypically characterized by forearm malformations. Recently identified role in Xenopus limb development and regeneration [24,25]. |
| ***Sh3glb1*** | -78% | Induction of Apoptosis | *Bax interacting factor* (*Bif1*) is a proapoptotic factor necessary for the regulation of outer mitochondrial membrane morphology [26]. |
| ***Shmt1*** | +99% | Glycine hydroxymethyl transferase activity | Polymorphisms in *Shmt1* implicated in Acute Lymphocytic Leukemia [27]. |
| ***Tcf4*** | --- | Transcriptional Regulation | bHLH transcription factor involved in repression of brain specific Fgf1 [28]. |
| ***Tdh*** | +100% | l-threonine dehydrogenase activity | Aberrantly methylated in breast cancer [29]. |
| ***Tdrd7*** | -95% | Nucleic Acid Binding | Tudor domain containing protein which interacts with Pictaire 2 to facilitate terminal differentiation of neurons. Tudor domains play a role in diverse cellular functions such as response to DNA strand breaks [30,31]. |
| ***Trp53*** | +90% | DNA Damage Response; Transcriptional Regulation | Cell cycle regulation with divergent roles in growth arrest or apoptosis dependent upon cellular context. Mutated or inactivated in approximately 60% of human tumors. Recent role described for Trp53 in the differentiation of ESCs via transcriptional repression of Nanog [32]. |

1. Sam M, Wurst W, Kluppel M, Jin O, Heng H, et al. (1998) Aquarius, a novel gene isolated by gene trapping with an RNA dependent RNA polymerase motif. Develpmental Dynamics 212: 304-317.

2. Stein P, Svoboda P, Anger M, Schultz RM (2003) RNAi: Mammalian oocytes do in without RNA-dependent RNA polymerase. RNA 9: 187-192.

3. Wang J, Zhou Y, Yin B, Du G, Huang X, et al. (2001) Ash2l: alternative splicing and downregulation during induced megakaryocytic differentiation of multipotential leukemia cell lines. Journal of Molecular Medicine 79: 399-405.

4. Molofsky AV, Pardal R, Iwashita T, Park I-K, Clarke MF (2003) Bmi-1 dependence distinguishes neural stem cell self-renewal from progenitor proliferation. Nature 425: 962-967.

5. Lessard J, Sauvageau G (2003) Bmi-1 determines the proliferative capacity of normal and leukaemic stem cells. 423: 255-260.

6. Wang Y, Cortez D, Yazdi P, Neff N, Elledge SJ, et al. (2000) BASC, a super complex of BRCA1-associated proteins involved in the recognition and repair of aberrant DNA structures. Genes Dev 14: 927-939.

7. Yoo NJ, Lee JW, Kim YJ, Soung YH, Kim SY, et al. (2004) Loss of caspase-2, -6 and -7 expression in gastric cancers. APMIS 112: 330-335.

8. Tetzlaff MT, Bai C, Finegold M, Wilson J, Harper JW, et al. (2004) Cyclin F Disruption Compromises Placental Development and Affects Normal Cell Cycle Execution. Mol Cell Biol 24: 2487-2498.

9. Eichmuller S, Usener D, Dummer R, Stein A, Thiel D, et al. (2001) Serological detection of cutaneous T-cell lumphoma-associated antigens. Proceedings of the National Academy of Sciences 98: 629-634.

10. Yoshima T, Yura T, Yanagi H (1998) Novel testis-specific protein that interacts with heat shock factor 2. Gene 214: 139-146.

11. Arenkiel BR, Tvrdik P, Gaufo GO, Capecchi MR (2004) Hoxb1 functions in both motoneurons and in tissues of the periphery to establish and maintain the proper neuronal circuitry. Genes Dev 18: 1539-1552.

12. Ioannidis P, Mahaira LG, Perez SA, Gritzapis AD, Sotiropoulou PA, et al. (2005) CRD-BP/IMP1 Expression Characterizes Cord Blood CD34+ Stem Cells and Affects c-myc and IGF-II Expression in MCF-7 Cancer Cells. J Biol Chem 280: 20086-20093.

13. Takeuchi T, Kojima M, Nakajima K, Kondo S (1999) Jumonji gene is essential for the neurulation and cardiac development of mouse embryos with a C3H/He Background. Mechanisms of Development 86: 29-38.

14. Bhagavatula MRK, Fan C, Shen G-Q, Cassano J, Plow EF, et al. (2004) Transcription factor MEF2A mutations in patients with coronary artery disease. Hum Mol Genet 13: 3181-3188.

15. Kim MY, Mauro S, Gevry N, Lis JT, Kraus WL (2004) NAD+-Dependent Modulation of Chromatin Structure and Transcription by Nucleosome Binding Properties of PARP-1. Cell 119: 803-814.

16. Fusaro G, Dasgupta P, Rastogi S, Joshi B, Chellappan S (2003) Prohibitin Induces the Transcriptional Activity of p53 and Is Exported from the Nucleus upon Apoptotic Signaling. J Biol Chem 278: 47853-47861.

17. Manjeshwar S, Branam DE, Lerner MR, Brackett DJ, Jupe ER (2003) Tumor Suppression by the Prohibitin Gene 3'Untranslated Region RNA in Human Breast Cancer. Cancer Res 63: 5251-5256.

18. Ohta H, Sawada A, Kim JY, Tokimasa S, Nishiguchi S, et al. (2002) Polycomb Group Gene rae28 Is Required for Sustaining Activity of Hematopoietic Stem Cells. J Exp Med 195: 759-770.

19. Shirai M, Osugi T, Koga H, Kaji Y, Takimoto E, et al. (2002) The Polycomb-group gene Rae28 sustains Nkx2.5/Csx expression and is essential for cardiac morphogenesis. J Clin Invest 110: 177-184.

20. de The H, Lavau C, Marchio A, Chomienne C, Degos L, et al. (1991) The PML-RAR[alpha] fusion mRNA generated by the t(15;17) translocation in acute promyelocytic leukemia encodes a functionally altered RAR. Cell 66: 675-684.

21. Ballas N, Grunseich C, Lu DD, Speh JC, Mandel G (2005) REST and Its Corepressors Mediate Plasticity of Neuronal Gene Chromatin throughout Neurogenesis. Cell 121: 645-657.

22. Westbrook TF, Martin ES, Schlabach MR, Leng Y, Liang AC, et al. (2005) A Genetic Screen for Candidate Tumor Suppressors Identifies REST. Cell 121: 837-848.

23. Akasaka T, Takahashi N, Suzuki M, Koseki H, Bodmer R, et al. (2002) MBLR, a new RING finger protein resembling mammalian Polycomb gene products, is regulated by cell cycle-dependent phosphorylation. Genes to Cells 7: 835-850.

24. Borozdin W, Boehm D, Leipoldt M, Wilhelm C, Reardon W, et al. (2004) SALL4 deletions are a common cause of Okihiro and acro-renal-ocular syndromes and confirm haploinsufficiency as the pathogenic mechanism. J Med Genet 41: e113-.

25. Neff AW, King MW, Harty MW, Nguyen T, Calley J, et al. (2005) Expression of Xenopus XISALL4 during limb development and regeneration. Develpmental Dynamics 233: 356-367.

26. Karbowski M, Jeong S-Y, Youle RJ (2004) Endophilin B1 is required for the maintenance of mitochondrial morphology. J Cell Biol 166: 1027-1039.

27. Skibola CF, Smith MT, Hubbard A, Shane B, Roberts AC, et al. (2002) Polymorphisms in the thymidylate synthase and serine hydroxymethyltransferase genes and risk of adult acute lymphocytic leukemia. Blood 99: 3786-3791.

28. Liu Y, Ray SK, Yang X-Q, Luntz-Leybman V, Chiu I-M (1998) A Splice Variant of E2-2 Basic Helix-Loop-Helix Protein Represses the Brain-specific Fibroblast Growth Factor 1 Promoter through the Binding to an Imperfect E-box. J Biol Chem 273: 19269-19276.

29. Miyamoto K, Fukutomi T, Akashi-Tanaka S, Hasegawa T, Asahara T, et al. (2005) Identification of 20 genes aberrantly methylated in human breast cancers. International Journal of Cancer 116: 407-414.

30. Hirose T, Kawabuchi M, Tamaru T, Okumura N, Nagai K, et al. (2000) Identification of tudor repeat associator with PCTAIRE 2 (Trap): A novel protein that interacts with the N-terminal domain of PCTAIRE 2 in rat brain. Eur J Biochem 267: 2113-2121.

31. Stucki M, Jackson SP (2004) Tudor domains track down DNA breaks. Nature Cell Biology 6: 1150-1152.

32. Lin T, Chao C, Saito Si, Mazur SJ, Murphy ME, et al. (2005) p53 induces differentiation of mouse embryonic stem cells by suppressing Nanog expression. Nature Cell Biology 7: 165-171.
